# Changes in lifespace and participation in community‐based occupations of people with acquired brain injury: A mixed methods exploration 6 months following occupational therapy driving assessment

**DOI:** 10.1111/1440-1630.70017

**Published:** 2025-04-13

**Authors:** Louise Bassingthwaighte, Louise Gustafsson, Matthew Molineux

**Affiliations:** ^1^ Discipline of Occupational Therapy, School of Health Sciences and Social Work Griffith University Brisbane Queensland Australia; ^2^ Occupational Therapy Department Princess Alexandra Hospital Brisbane Queensland Australia; ^3^ School of Health Sciences and Social Work Griffith University Brisbane Queensland Australia

**Keywords:** acquired brain injury, community participation, lifespace, occupation, occupational choice, occupational identity, occupational therapy driver assessment

## Abstract

**Introduction:**

Changes arising from acquired brain injury may influence how individuals engage in valued community‐based occupations such as driving. ‘Lifespace’ describes the area within which a person lives their life and represents opportunity for participation in out‐of‐home occupations. This study explored lifespace trajectory from pre‐ to 6 months post‐occupational therapy driver assessment, to understand how, why, where, and with whom access and participation in community‐based occupations is influenced by assessment outcome.

**Methods:**

Adults with acquired brain injury referred for occupational therapy driver assessment were recruited to the mixed methods study involving a travel diary, lifespace assessment, and semi‐structured interviews. Qualitative analysis was guided by interpretive description.

**Consumer and Community Involvement:**

No consumer and community involvement

**Results:**

Overall, 38 participants (55.3% male) aged 26 to 65 years reported increased lifespace 6 months following the conduct of an occupational therapy driver assessment. There was increased engagement in leisure pursuits (175%), work (23%), and social participation (21%) with reduced participation in health management (−50%) and instrumental activities of daily living (−15.4%) occupations post‐OTDA. However, lifespace was significantly related to driver status, with those who had returned to driving more likely to access their community with greater frequency and less support (*p* < 0.001). Non‐drivers experienced a deteriorating restricted lifespace. Analysis of semi‐structured interviews (n = 12) created three broad themes that largely differed according to driver status: (i) ‘Being me’—reconstructing occupational identity, (ii) opportunities for participation and the influence of choice, and (iii) ‘Having connection’ and impacts on wellbeing.

**Conclusion:**

Driver status influences the trajectory of lifespace following participation in an occupational therapy driver assessment after acquired brain injury. Drivers experienced increased lifespace with greater opportunities to control engagement in community‐based occupations with flexibility and spontaneity. Non‐drivers reported diminished lifespace and occupational participation trajectories and require further support to facilitate occupational adaptation to increase opportunities for engagement in away‐from‐home occupations.

**PLAIN LANGUAGE SUMMARY:**

After an acquired brain injury (ABI), many people find it harder to go out and do activities away from home. A common change is losing the ability to drive. An occupational therapy driver assessment (OTDA) checks if someone is ready to drive again. This study looked at how getting back to driving, or not, affected involvement in community activities. People who returned to driving reported doing more activities, more often, and with less help. They spent more time on leisure, work, and social activities. Those who did not drive went out less, visited fewer places, and relied more on others. When they did go out, it was mostly for essential tasks like shopping and health appointments. For those not able to drive, extra services and supports are needed to help build skills. This is the first study to look at how driving is connected to taking part in community activities after a brain injury. More research is needed to confirm findings.

**Key Points for Occupational Therapy**

- Return to driving post‐ABI increases engagement in community‐based occupations.
- A ‘not‐fit‐to‐drive’ OTDA outcome may signal need for additional supports to enhance skills, routines, and habits that
  ○ initiate and sustain engagement in community‐based occupations and
  ○ facilitate control over participation choices.
- Exploration of non‐engagement in community‐based occupations post‐ABI is required.

## 1 INTRODUCTION

Acquired brain injury (ABI) arises from non‐congenital damage to the brain that is unrelated to a degenerative disease (Wallis et al., 2022). Stroke, traumatic brain injury (TBI), and cerebral infection are just a few causes of ABI, which manifest in an array of physical, cognitive, behavioural, and psychological sequelae (Ponsford et al., 2014; Smith et al., 2018). With the global prevalence of TBI reported at 48.99 million (Lucchesi et al., 2019) and 15 million new strokes recorded annually worldwide (Feigin et al., 2022; World Health Organization, 2020), ABI is a leading cause of life‐long disability (Roozenbeek et al., 2013).

Beyond prevalence, consideration is required of the consequences arising from ABI. The World Health Organization defines ‘participation’ as ‘involvement in a life situation’ (World Health Organization, 2001, p. 24), whereas ‘occupational participation’ is a concept that encompasses the interactions between the person, their occupation, and their environment (Egan & Restall, 2022). Individually and collectively, these elements influence capacity to access, initiate, and sustain participation in valued occupations such as shopping, driving a car, volunteering, and social pursuits (Chang et al., 2020; Egan & Restall, 2022; Kersey et al., 2020). Occupational participation reflects not only involvement in and performance of meaningful endeavours but also the level of satisfaction gained from engagement (Egan & Restall, 2022). Adverse post‐ABI changes may influence how, when, or if the person is able to engage in occupations that provide a sense of purpose, achievement, or belonging (Ikiugu et al., 2019; Klepo et al., 2020; Travers et al., 2016). Diminished occupational participation has been established following ABI, with community‐based initiatives most frequently constrained (Blömer et al., 2015; Kersey et al., 2020; Lee et al., 2015). Consequently, compromised community integration, reduced quality of life, increased social isolation, and greater depressive symptoms may be experienced contributing to changes in the person's occupational identity (Blömer et al., 2015; Bryson‐Campbell et al., 2013; George et al., 2019).

Occupational disruption may transpire when changes and disturbances to usual patterns of occupational engagement and performance occur (Molineux, 2017). Following ABI, one community‐based occupation that is frequently disrupted is driving (Erler et al., 2018; Liddle et al., 2012). Driving is a complex occupation where safe and competent performance is reliant on accurate and timely perception, interpretation of, and response to dynamic and often unpredictable environments. Many jurisdictions have risk mitigation processes, such as medical guidelines and Occupational Therapy Driver Assessments (OTDA), to ascertain the impact of ABI‐related impairments on readiness to return to driving and driving performance (National Transport Commission, 2022). In Australia, OTDA are conducted by occupational therapists with post‐graduate qualifications. Comprehensive assessments occur following a medical practitioner referral and incorporate off‐road evaluations and on‐road driving components (Fields et al., 2018). Whereas medical guidelines and assessment processes are established in Australia, access to services is not universally available. Barriers include inadequate provision of return to driving information (Chua et al., 2012; Fleming et al., 2014), limited availability of driving‐specific services (Dun et al., 2015), and costs associated with private provider OTDA (Kay et al., 2012). ABI recovery and risk mitigation pathways can therefore be lengthy, contributing to a protracted period of non‐driving (Erler et al., 2018; Fleming et al., 2014). As driving is an enabler of engagement in away‐from‐home occupations, driving disruption following ABI restricts community reintegration in terms of access, opportunities for social connection, and occupational participation (Bassingthwaighte et al., 2025; Griffen et al., 2009; Liddle et al., 2012). The period of driving disruption following ABI may be a time of occupational adaptation, when the person and their support network adjust and modify prior routines, habits, and choices and/or create new opportunities to facilitate engagement and participation in away‐from‐home occupations.

One approach to understanding the extent to which a person adapts to their ABI and re‐engages in their community is through the exploration of ‘lifespace’. This term refers to the geographical area within which a person conducts their life and describes the frequency and extent to which individuals enter their community (Liddle et al., 2014; Yang et al., 2017). The concept of lifespace emerged as a measure of community movement for older persons (Baker et al., 2003). It has been found to reflect wellbeing, health, and mobility following stroke (Johnson et al., 2020; Yang et al., 2017), noted to reduce when retiring from driving in older adults (Phillips et al., 2019), and has been used to describe the impact of TBI on family members (Liang et al., 2016). Lifespace has been used to investigate the period of driving disruption following the onset of ABI, at a time immediately prior to the conduct of an OTDA (Bassingthwaighte et al., 2025). This period was confirmed as a time of restricted community access that increased dependence on support networks, demanded occupational adaptation in order to engage and participate in community‐based occupations, and changed occupational identity. However, it was unclear whether these findings were indicative of the consequences of the ABI, a reflection of the influence of driving disruption or a combination of these factors. Furthermore, the influence the OTDA process and outcomes on lifespace and participation following ABI are unknown.

This study first aimed to explore the trajectory, or description of how lifespace changes, from pre‐ to post‐OTDA for participants with an ABI. Second, the study aimed to understand how access and participation in community‐based occupations changed from pre‐ to post‐OTDA following ABI in terms of how, why, where, and with whom, with consideration given to driver status. Finally, the study aimed to explore the impact of driving status on lifespace following ABI.

## 2 MATERIALS AND METHODS

### 2.1 Study design

**FIGURE 1:**
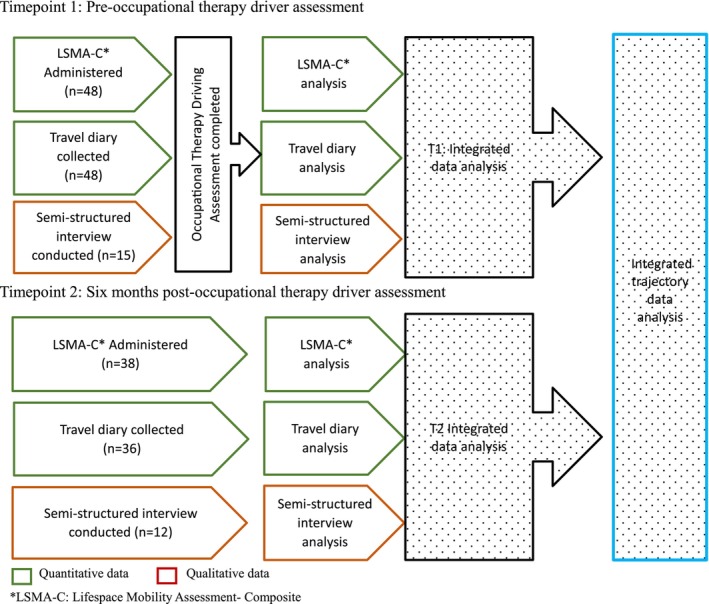
Study design and participant flow.

A mixed methods, convergent research design was used to explore the influence of the OTDA process and outcomes on the trajectory of lifespace following ABI as illustrated in Figure 1. The study protocol was approved by the health service (HREC/2019/QMS/57505) and university (GU2020/115) ethics committees and enabled learnings from varied methods of investigation to be corroborated to enhance confidence in findings (Creswell & Creswell, 2018; Timans et al., 2019). Timepoint 1 (T1) reflected the period of driving disruption following ABI when a referral for OTDA had been made but was yet to be conducted. Methodology and findings from T1 have been previously reported (Bassingthwaighte et al., 2025). Timepoint 2 (T2) occurred 6 months after the conduct of an OTDA when participants had time to experience consequences of three potential outcomes: (i) fit to drive, (ii) not fit to drive, or (iii) on‐road driving remediation. This paper reports on T2 findings and the trajectory or change between T1 and T2.

### 2.2 Study setting

The research was undertaken at an Australian tertiary public hospital providing multi‐disciplinary services throughout the continuum from intensive and acute care, through rehabilitation, into outreach services for people following ABI. Services included an outpatient occupational therapy driver assessment and rehabilitation programme.

### 2.3 Participant selection and eligibility criteria

Adults referred for OTDA following the onset of ABI were the target of this study. Inclusion criteria were: (a) aged between 18 years and 65 years inclusive; (b) medically stable and cleared by a doctor to participate in OTDA; (c) holder of a current and valid provisional or open driver's licence; and (d) diagnosis of ABI. Being a learner driver and/or having had a previous neurological condition/incident excluded participation in the study. Recruitment commenced May 2021 and ceased November 2022, with T2 follow‐up concluding in July 2023.

### 2.4 Sampling strategy

A mixed methods sampling strategy was adopted. For quantitative data, all participants recruited to the study represented a homogenous sample (i.e., all had been referred for OTDA following ABI). Different purposive sampling methods were adopted for the pre‐ and post‐OTDA qualitative elements. Whereas every third participant was interviewed pre‐OTDA, post‐OTDA, maximum variation sampling based on T1 OTDA outcome was used to select the first four participants recommended as fit to drive, not fit to drive, or not fit to drive but suitable for on‐road driving remediation, to participate in a semi‐structured interview at T2 (Patton, 2015). This pragmatic strategy endeavoured to obtain diverse representation across the three possible OTDA outcomes to enhance credibility and to manage the number of interviews being conducted by the clinician researcher (Thorne, 2017).

### 2.5 Methods of data collection

#### 2.5.1 Demographic and diagnostic data

Demographic and diagnosis‐related data were extracted from electronic medical records.

#### 2.5.2 Quantitative measures

**FIGURE 2:**
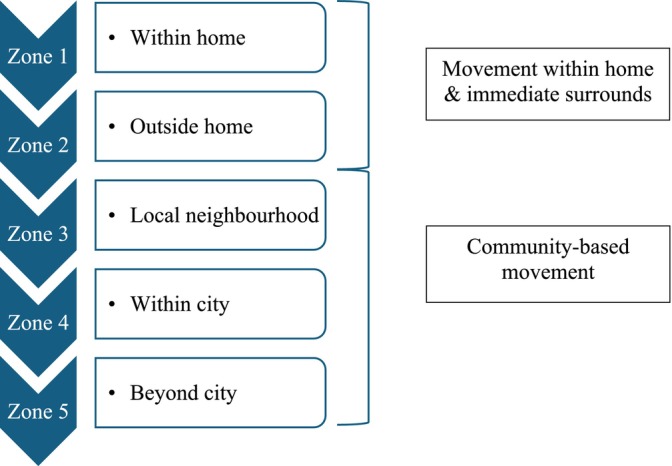
Lifespace zones.

Two quantitative measures were used. First, a designed‐for‐purpose travel diary captured information about the frequency and purpose of travel, transport mode, and support requirements (see Appendix S1). To enhance the consistency of completion, prior to administration, participants were given a verbal overview of completion requirements, the travel diary was accompanied by a concise written guideline and participants had opportunities to discuss any queries as they arose or during a phone call from the first author during the completion phase. Testing of the accuracy of completion did not occur. The second quantitative measure involved the administration of Lifespace Mobility Assessment–Composite (LSMA‐C) (Baker et al., 2003; Phillips et al., 2019). Confirmed as a valid and reliable measure of community mobility for older adults (Baker et al., 2003; Kuspinar et al., 2023), the LSMA‐C quantifies the frequency and level of independence participants accessed five increasingly distant geographical zones represented in Figure 2 over a 4‐week period of time to determine a lifespace score ranging from 0 to 120 (Johnson et al., 2020; Phillips et al., 2019). A score below 60 represented restricted lifespace (Phillips et al., 2019).

#### 2.5.3 Qualitative data collection

Semi‐structured interviews were conducted to gather qualitative data. The interview guide at T2 (see Table 1) differed from the T1 interview guide to enable participants to reflect on their OTDA outcomes and contemplate the influence of driving status on current participation in community‐based occupations.

**TABLE 1:** T2 interview guide.

Question	Potential prompts
To start with, can you tell me about your occupational therapy driving assessment process and experience?	What was the outcome of the initial assessment? How did you feel about the outcome at the time? Was the outcome different from what you expected? How did you find driving lessons? Are there any areas you feel still need some work? (if appropriate)
This interview is about understanding your movement around your community, including where and why you go there. Thinking about the time since your occupational therapy driving assessment, can you talk me through an average week?	Is there anything different about how you get about now compared to before your OTDA? Has anything changed over the past 6 months with respect to how often you leave the house, where you travel to, how you travel and with whom, etc.?
Now, if we can look at your travel diary, can you tell me about these trips?	Explore meaning/purpose, experience, barriers/enablers. Has it been a typical few weeks for you (e.g., illness, unusual travel, holidays)?
Over the past 6 months, has your driving status changed anything about what you do outside your home?	Are there any activities you have recommenced/started or stopped engaging in? Are there any different ways of getting about that you use now compared to before your driving assessment or before your injury/stroke?
Knowing what you do now, is there anything that you would tell yourself back during that time when you were waiting to start the return to driving process?	Do you have any advice for people who may be leaving hospital and are unsure about their return to driving pathway?
Thinking about how you move around in your community now, what do you think are the important things that health professionals should know?	Prompts: changes, positives, negatives, what we need to do differently, etc.

### 2.6 Post‐OTDA procedures

#### 2.6.1 Quantitative elements

Participants were mailed customised day‐per‐page travel diary sheets with a concise completion guideline. Undertaken over a 2‐week period, at least 6 months following completion of OTDA, participants completed daily entries concerning the purpose, frequency, extent of travel, mode of transport and support needs when leaving home. Participants either brought their completed travel diary to a scheduled appointment at the study centre or mailed the document via replied‐paid envelope. Results were coded to calculate frequencies. The LSMA‐C was administered based on participant self‐report, either in person or over the telephone.

#### 2.6.2 Qualitative element

Depending upon participant preference, semi‐structured in‐depth interviews were conducted by the first author at the same appointment scheduled to collect quantitative data or via telephone to gain context and enhance understanding of issues impacting participation in occupations conducted outside the home. Data collection and analysis methodology was guided by interpretive description (Thorne, 2017). This approach is often used when seeking to understand experiences with an aim to influence clinical practices (Thompson Burdine et al., 2021). It shaped the interviews and analysis as there was a focus on the experiences of occupations conducted outside the home in order to inform occupational therapy ABI rehabilitation practice.

Interviews were audio recorded and transcribed by a professional transcription service. Transcription accuracy was verified by the first author prior to the commencement of data analysis. QSR International NVivo (Release 1.7) was used to manage the coding process for each individual transcript and then to assemble, explore and construct themes in an iterative manner. All authors contributed to the analysis.

### 2.7 Data analysis

#### 2.7.1 Quantitative analysis

To address the first two aims, descriptive statistics were used to understand travel diary and LSMA‐C data. ‘Community trips’ represented the number of occasions the participant left their home and ‘trip legs’ were components of each trip. For instance, when a participant left home to meet a friend for lunch and then attended the local library before returning home, one community trip involving three trip legs were counted (home to lunch; lunch to library; library to home). The Occupational Therapy Performance Framework 4th Edition (OTPF 4th Ed) classification of occupations was used to assist the classification of the purpose of travel in the analysis (Boop et al., 2020). To explore the final aim of the study, a point‐biserial correlation was run between driving status and LSMA‐C total score at T2 using IBM SPSS Statistics version 29 (Laerd Statistics, 2017). Normal distribution was evaluated with the Shapiro–Wilk test and homogeneity of variances was assessed by Levene's test for equality of variances.

#### 2.7.2 Qualitative analysis

The qualitative element of the research contributed to the exploration of the first two aims. Interpretive description guided the analysis of interview transcripts to generate an understanding of lived experiences with the intent to inform occupational therapy clinical practice (Thompson Burdine et al., 2021; Thorne, 2017). Confirmation of transcript accuracy preceded immersion in transcripts to comprehend data. Synthesis and reflection on meaning initially occurred through inductive coding of individual transcripts and then by collective consideration by all authors to synthesise meaning. Coding was conducted iteratively over five rounds, progressing from concrete codes through increasingly interpretive analysis to conceptualise relationships and define themes before being recontextualising into findings.

Qualitative findings were reported in terms of driver status at T2. ‘Drivers’ represented those deemed fit to drive at T1 OTDA and those who achieved a fit to drive recommendation following on‐road driving remediation. ‘Non‐drivers’ included participants who were recommended not fit to drive either at T1 OTDA or following on‐road driving remediation.

#### 2.7.3 Trustworthiness

A number of processes and actions aimed to enhance trustworthiness and credibility. First, epistemological integrity was maintained with a pragmatic philosophical paradigm guiding the creation of knowledge in this study (Creswell & Creswell, 2018). Arising from a desire to create actionable knowledge to solve complex real‐world concerns, this approach addressed all study aims by exploring if OTDA outcome enhanced, restricted, or had no impact upon where, how, why, and with whom access and participation in community‐based occupations occurred following ABI. Triangulation of data sources aimed to enhance representative credibility and an audit trail summarised analytic logic. Finally, to illustrate interpretive authority, author positionality, member checking during interviews, and direct quotes were used to contextualise constructed truths (LaCroix, 2022; Thorne, 2017).

#### 2.7.4 Integrated analysis

Systematic integrative procedures advocated by Creswell and Plano Clark (2018) and Johnson et al. (2017) were used to integrate data. At each timepoint, findings from quantitative and qualitative arms were analysed independently and then considered as a whole through a process of triangulation to explore the concepts of lifespace and occupational participation. A table was used to merge data, to combine and jointly display qualitative and quantitative findings (Johnson et al., 2017). Between timepoint comparisons were then conducted to explore the trajectory of lifespace from pre‐ to post‐OTDA. For quantitative data, this involved T1 to T2 percentage change calculations. For qualitative data, theme comparison with consideration to driver status was explored.

### 2.8 Positionality Statement

The research team was comprised of clinical and academic occupational therapists. The first author has extensive expertise in exploring capacity to return to driving following ABI, perceiving return to driving as an important goal for many following acquired injury and valuing engagement in away‐from‐home occupations. Two authors have experience as clinicians and researchers working with people with ABI. One member of the research team was less familiar with the clinical area yet highly experienced in qualitative research methodology and challenged assumptions to mitigate bias throughout the design, analysis, and interpretation phases. All authors collaborated to develop the concept, methodological design, prepare the travel diary, and interview schedule and analyse data.

## 3 RESULTS

### 3.1 Quantitative findings

Table 2 presents demographic, diagnostic, and driving‐related characteristics. Thirty‐eight (79.2%) of the 48 T1 participants contributed at T2. Aged between 26 and 65 years, most participants were male (55.3%), born in Australia (63.2%), and resided in urban locations (92.2%). Demographic characteristics and diagnostic groups largely reflected T1 proportions, with stroke most frequently represented (n = 17, 44.7%) followed by aneurysm (10, 26.4%) and TBI (5, 13.2%). Median time since onset was 390 days, and on average, participants had 31.2 years of driving experience. Thirty‐one (81.6%) participants were drivers at T2 post‐OTDA evaluation.

**TABLE 2:** Demographic, diagnostic, and driving‐related characteristics.

		T1: Pre‐OTDA (n = 48)	T2: Post‐OTDA (n = 38)
Age, years mean (median, range)		50.2 (52, 26–65)	50.5 (52, 26–65)
Gender, n (%)			
Male		27 (56.3)	21 (55.3)
Female		21 (43.8)	17 (44.7)
Cultural identity			
Aboriginal		1 (2.1)	0 (0)
Other		47 (97.9)	38 (100)
Country of birth, n (%)			
Australia		31 (64.6)	24 (63.2)
Foreign (England, New Zealand, Vietnam, Malaysia, Netherlands, Papua New Guinea, Republic of South, Korea, Samoa, South Africa, Thailand, the United States of America, Ghana)		17 (35.4)	14 (36.8)
Community location, n (%)			
Urban	Inner metropolitan	23 (47.9)	16 (42.2)
	Outer metropolitan	21 (43.8)	19 (50.0)
Regional	Inner regional	3 (6.3)	2 (5.3)
	Outer regional	1 (2.1)	1 (2.6)
Diagnosis, n (%)			
Stroke		22 (45.8)	17 (44.7)
Aneurysm		12 (25.0)	10 (26.4)
Traumatic brain injury		7 (14.5)	5 (13.2)
Brain infection		3 (6.3)	3 (7.9)
Tumour		2 (4.2)	1 (2.6)
Carbon monoxide poisoning		1 (2.1)	1 (2.6)
Hypoxic brain injury		1 (2.1)	1 (2.6)
Severity on admission, n (%)			
Stroke: NHISS			
Mild (1–4)		8 (36.4)	8 (47.1)
Mild to moderately severe (5–14)		9 (40.9)	5 (29.4)
Missing data		5 (22.7)	4 (23.5)
Aneurysm: WFNS			
Grade 1		5 (41.7)	4 (40)
Grade 2		3 (25.0)	3 (30)
Grade 3		0 (0)	0 (0)
Grade 4		1 (8.3)	0 (0)
Missing data		3 (25.0)	3 (30)
PTA, days mean (range)		9.5 (3–27)	11.7 (3–27)
Moderate (1–7 days)		3 (42.9)	2 (40)
Severe (1–4 weeks)		2 (28.6)	1 (20)
Missing data		2 (28.6)	2 (40)
Time since onset, days mean (median, range, IQ range)		666 (390, 34–7566, 163–715)	678 (390, 34–7566, 182–643)
Highest level of education, n (%)			
Did not complete school		17 (35.4)	14 (36.8)
Completed high school		11 (22.9)	6 (15.8)
TAFE/technical college		4 (8.3)	4 (10.5)
Degree		13 (27.1)	12 (31.6)
Postgraduate degree		3 (6.3)	2 (5.3)
Occupational group pre‐ABI, n (%)			
Manger		1 (2.1)	0 (0)
Professional		14 (29.2)	12 (31.6)
Technicians and trade		3 (6.3)	3 (7.9)
Community and personal service		4 (8.3)	4 (10.6)
Clerical and administrative		2 (4.2)	2 (5.3)
Sales		2 (4.2)	0 (0)
Machinery operators and drivers		7 (14.5)	6 (15.7)
Labourers		9 (18.7)	6 (15.7)
Other—student, volunteer, unemployed, home duties		6 (12.5)	5 (13.2)
Driving experience			
Experience, years mean (range)		30.9 (2–46)	31.2 (2–46)
Driving status			
Not fit to drive		48 (100)	7 (18.4)
Fit to drive		0 (0)	31 (81.6)
No car accidents^a^, n (%)		38 (79.2)	38 (100%)
No traffic infringements^a^, n (%)		36 (75.0)	38 (100%)

^a^ Frequency of car accidents or traffic infringements was measured by self‐report over the 5‐year period pre‐OTDA (T1) and for the intervening 6 months at T2.

#### 3.1.1 LSMA‐C

Average total LSMA‐C scores increased from 60.8 (SD: 17.6) pre‐OTDA to 73.3 (SD:21.1) post‐OTDA. Participants reporting a restricted lifespace (i.e., LSMA‐C ≤ 60) reduced from 54.2% at T1 to 28.9% at T2.

#### 3.1.2 Lifespace and driver status

**FIGURE 3:**
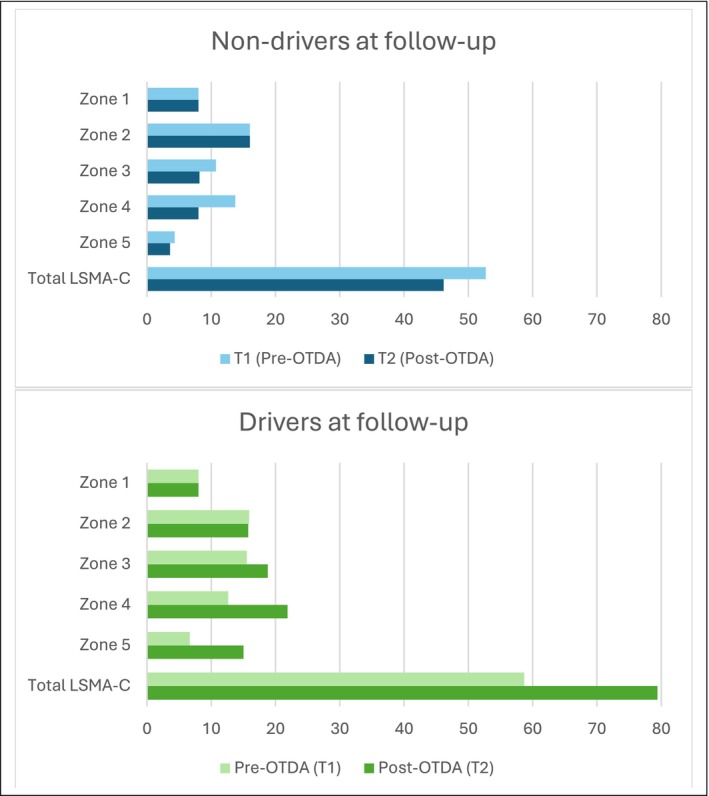
Lifespace trajectory based on driver status at follow‐up.

**FIGURE 4:**
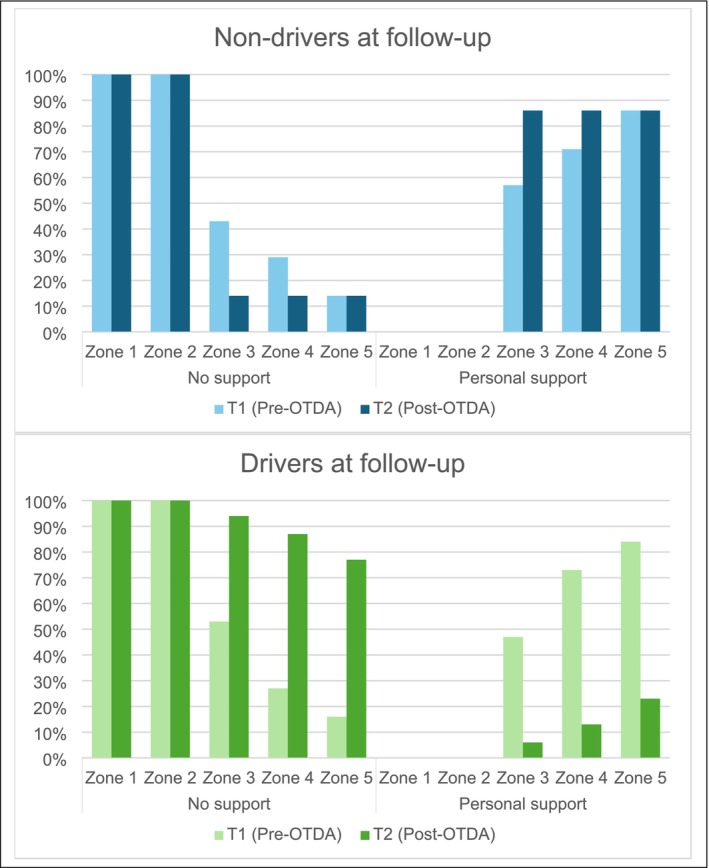
Lifespace support levels based on driver status at follow‐up.

At T2, 100% of non‐drivers experienced restricted lifespace compared to 19.3% of drivers. A point‐biserial correlation was run between driving status and lifespace score. Preliminary analyses assessed by boxplot showed a single outlier in the driving group. A decision was made to proceed with the analysis with the outlier included, as the single outlier was not extreme and was below the mean, suggesting any impact would understate the influence of driving status on lifespace score. A second analysis was conducted with the outlier excluded to explore influence. Negligible impact was observed with significance, increasing confidence in results (see Appendix S2 for comparative results with and without outlier inclusion). There was a strong, statistically significant correlation between driving status and lifespace score, *r*_pb_(36) = 0.620, *p* < 0.001, with drivers reporting greater lifespace (mean = 79.4, SD = 17.8) than non‐drivers (mean = 46.1, SD = 9.8). Driving status accounted for 38.4% of the variability in lifespace scores. Figure 3 displays LSMA‐C total and zones score trajectories based upon driver status at T2. The requirement for personal support or otherwise to achieve lifespace at T1 and T2 is illustrated in Figure 4.

#### 3.1.3 Travel diary

Table 3 illustrates the travel diary findings inclusive of trajectory from T1 to T2 overall as a cohort and according to driver status at T2 in the form of percentage change. Unless otherwise stated, the findings below relate to the trajectory of all participants irrespective of driver status at T2.

**TABLE 3:** Trajectory of travel diary findings by assessment point and driver status.

	T1 (Pre‐OTDA) (n = 48)	T2 (Post‐OTDA) (n = 36)	Trajectory^a^
Travel frequency			
Number of days^b^, n	672	504	
Drivers at follow‐up^c^	406	406	
Non‐drivers at follow‐up^d^	98	98	
Number of times left home/day^b^ ^,^ ^e^, n (mean, SD)	707 (1.1, 0.9)	589 (1.2, 1.1)	↑ 11.4%
Drivers at follow‐up^c^	397 (1.0, 0.8)	486 (1.2, 1.0)	↑ 22.4%
Non‐drivers at follow‐up^d^	81 (0.8, 0.9)	103 (1.1, 1.2)	↑ 26.5%
Total number of trip legs^b^ ^,^ ^f^, n (mean, SD)	1474 (2.2, 2.0)	1209 (2.4, 2.2)	↑ 9.6%
Drivers at follow‐up^c^	840 (2.1, 1.8)	1018 (2.5, 2.3)	↑ 21.3%
Non‐drivers at follow‐up^d^	176 (1.8, 2.1)	191 (2.0, 2.1)	↑ 8.3%
Total day trips^b^ ^,^ ^g^, n (mean, SD)	1272 (1.9, 0.2)	1072 (2.1, 2.0)	↑12.7%
Drivers at follow‐up^c^	740 (1.8, 1.7)	886 (2.2, 2.1)	↑ 19.8%
Non‐drivers at follow‐up^d^	161 (1.6, 2.0)	186 (1.9, 2.0)	↑ 15.9%
Total night trips^b^ ^,^ ^h^, n (mean, SD)	202 (0.3, 0.8)	134 (0.3, 0.6)	↓12.9%
Drivers at follow‐up^c^	104 (0.3, 0.6)	129 (0.3, 0.7)	↑ 23.1%
Non‐drivers at follow‐up^d^	15 (0.2, 0.5)	5 (0.1, 0.3)	↓ 66.7%
Purpose of travel OTPF 4th edition			
Total number of occupations, n (mean, SD)	539 (15.0, 7.5)	610 (16.9, 8.3)	↑ 13.2%
Drivers at follow‐up^c^	448 (15.5, 6.4)	527 (18.2, 8.0)	↑ 17.6%
Non‐drivers at follow‐up^d^	91 (13.0, 11.7)	83 (11.9, 7.7)	↓ 8.8%
Leisure^b^, n (%)	58 (7.7)	129 (21.2)	↑175.3%
Drivers at follow‐up^c^	25 (5.6)	110 (20.9)	↑ 273.2%
Non‐drivers at follow‐up^d^	18 (19.8)	19 (23.5)	↑ 18.7%
Work^b^, n (%)	74 (9.9)	74 (12.2)	↑23.2%
Drivers at follow‐up^c^	34 (7.6)	73 (13.8)	↑ 81.6%
Non‐drivers at follow‐up^d^	5 (5.5)	1 (1.2)	↓ 78.2%
Social participation^b^, n (%)	126 (16.8)	124 (20.4)	↑21.4%
Drivers at follow‐up^c^	85 (19.0)	116 (22.0)	↑ 15.8%
Non‐drivers at follow‐up^d^	18 (19.8)	8 (9.9)	↓ 50.0%
IADL^b^ ^,^ ^g^, n (%)	291 (38.9)	200 (32.9)	↓15.4%
Drivers at follow‐up^c^	179 (40.0)	165 (31.3)	↓ 21.7%
Non‐drivers at follow‐up^d^	37 (40.7)	35 (43.2)	↑ 6.1
Health management^b^, n (%)	200 (26.6)	80 (13.2)	↓50.4%
Drivers at follow‐up^c^	124 (27.7)	62 (11.8)	↓ 57.4%
Non‐drivers at follow‐up^d^	25 (27.5)	18 (22.2)	↓ 17.1%
Education^b^, n (%)	1 (0.1)	1 (0.1)	No change
Drivers at follow‐up^c^	1 (0.1)	1 (0.1)	No change
Non‐drivers at follow‐up^d^	0 (0)	0 (0)	No change
Mode of transport, n (%)			
Private car^b^	323 (52.2)	314 (70.6)	↑ 35.2%
Drivers at follow‐up^c^	218 (58.0)	274 (71.9)	↑ 24.0%
Non‐drivers at follow‐up^d^	44 (61.1)	40 (61.5)	↑ 0.7%
Walking^b^	184 (29.7)	87 (19.6)	↓ 34.0%
Drivers at follow‐up^c^	104 (27.7)	71 (18.6)	↓ 32.9%
Non‐drivers at follow‐up^d^	13 (18.1)	16 (24.6)	↑ 35.9%
Public transport (bus, train, ferry)^b^	55 (8.9)	26 (4.9)	↓ 44.9%
Drivers at follow‐up^c^	21 (5.6)	18 (4.7)	↓ 16.1%
Non‐drivers at follow‐up^d^	6 (8.3)	8 (12.3)	↑ 48.2%
Ride share (taxi and Uber)^b^	39 (6.3)	16 (4.3)	↓ 31.7%
Drivers at follow‐up^c^	27 (7.2)	16 (4.2)	↓ 41.7%
Non‐drivers at follow‐up^d^	1 (1.4)	0 (0)	↓ 100.0%
Bicycle	13 (2.1)	0 (0.0)	↓ 100.0%
Drivers at follow‐up^c^	1 (0.3)	0 (0)	↓ 100.0%
Non‐drivers at follow‐up^d^	8 (11.1)	0 (0)	↓ 100.0%
Alternative transport (e.g., community flyer)	3 (0.5)	1 (0.3)	↓ 40.0%
Drivers at follow‐up^c^	3 (0.8)	1 (0.3)	↓ 62.5%
Non‐drivers at follow‐up^d^	0 (0)	0 (0)	No change
Other (plane, boat, ambulance)	2 (0.3)	2 (0.4)	↑ 33.3%
Drivers at follow‐up^c^	2 (0.5)	1 (0.3)	↓ 40.0%
Non‐drivers at follow‐up^d^	0 (0)	1 (1.5)	↑ Infinity
Company present on any trip leg, n (%)	331 (68)	187 (37)	↓ 45.6%
Drivers at follow‐up^c^	220 (54.2)	150 (36.9)	↓ 31.9%
Non‐drivers at follow‐up^d^	45 (45.9)	37 (35.9)	↓ 21.8%
Who? n (%)			
Partner	144 (31.9)	74 (28.5)	↓ 10.7%
Drivers at follow‐up^c^	89 (30.1)	67 (33.0)	↑ 9.6%
Non‐drivers at follow‐up^d^	15 (22.4)	7 (12.3)	↓ 45.1%
Dependent child/ren	77 (17.1)	72 (27.7)	↑ 62%
Drivers at follow‐up^c^	56 (18.9)	58 (28.6)	↑ 51.3%
Non‐drivers at follow‐up^d^	16 (23.9)	14 (24.6)	↑ 2.9%
Parent	71 (15.7)	28 (10.8)	↓ 31.2%
Drivers at follow‐up^c^	56 (18.9)	18 (8.9)	↓ 52.9%
Non‐drivers at follow‐up^d^	11 (16.4)	10 (17.5)	↑ 6.7%
Friend	40 (8.9)	44 (16.9)	↑ 89.9%
Drivers at follow‐up^c^	32 (10.8)	40 (19.7)	↑ 82.4%
Non‐drivers at follow‐up^d^	1 (1.5)	4 (7.0)	↑ 366.7%
Other family (e.g., child who drives, sibling)	70 (15.6)	25 (9.6)	↓ 38.5%
Drivers at follow‐up^c^	42 (14.2)	13 (6.4)	↓ 54.9%
Non‐drivers at follow‐up^d^	10 (14.9)	12 (21.1)	↑ 41.6%
Support worker	24 (5.2)	13 (5.0)	↓ 3.8%
Drivers at follow‐up^c^	11 (3.7)	4 (2.0)	↓ 45.9%
Non‐drivers at follow‐up^d^	13 (19.4)	9 (15.8)	↓ 18.6%
Professional driver	22 (4.9)	4 (1.5)	↓ 69.4%
Drivers at follow‐up^c^	20 (6.8)	3 (1.5)	↓ 77.9%
Non‐drivers at follow‐up^d^	1 (1.5)	1 (1.7)	↑ 13.3%
Type of support, n (%)			
Driver	276 (71.0)	28 (20.6)	↓ 71.0%
Drivers at follow‐up^c^	185 (75.2)	10 (9.9)	↓ 86.8%
Non‐drivers at follow‐up^d^	44 (65.7)	18 (51.4)	↓ 21.8%
Driver and other support	52 (13.4)	17 (12.5)	↓ 6.7%
Drivers at follow‐up^c^	32 (13.0)	8 (7.9)	↓ 39.2%
Non‐drivers at follow‐up^d^	11 (16.4)	9 (25.7)	↑ 56.7%
No support‐social presence	61 (15.7)	91(66.9)	↑ 326.1%
Drivers at follow‐up^c^	29 (11.8)	83 (82.2)	↑ 596.6%
Non‐drivers at follow‐up^d^	12 (17.9)	8 (22.9)	↑ 27.9%
Difficult of trip, n (%)			
Very easy	208 (39.5)	173 (47.1)	↑ 19.2%
Drivers at follow‐up^c^	135 (40.7)	160 (51.4)	↑ 26.3%
Non‐drivers at follow‐up^d^	12 (21.1)	13 (23.2)	↑ 10.0%
Easy	202 (38.4)	157 (42.8)	↑ 11.5%
Drivers at follow‐up^c^	114 (34.3)	121 (38.9)	↑ 13.4%
Non‐drivers at follow‐up^d^	27 (47.4)	36 (64.3)	↑ 35.7%
Not difficult or easy	74 (14.1)	19 (5.2)	↓ 63.1%
Drivers at follow‐up^c^	58 (17.5)	18 (5.8)	↓ 66.9%
Non‐drivers at follow‐up^d^	5 (8.8)	1 (1.8)	↓ 79.5%
Difficult	39 (7.4)	18 (4.9)	↓ 33.8%
Drivers at follow‐up^c^	22 (6.6)	12 (3.9)	↓ 40.9%
Non‐drivers at follow‐up^d^	13 (22.8)	6 (10.7)	↓ 53.1%
Very difficult	3 (0.6)	0 (0.0)	↓ 100%
Drivers at follow‐up^c^	0 (0)	0 (0)	No change
Non‐drivers at follow‐up^d^	0 (0)	0 (0)	No change

*Note*: No occasions recorded pre‐ or post‐OTDA for activities of daily living, rest/sleep or play.

Abbreviation: IADL, instrumental activities of daily living.

^a^ Trajectory calculated as percentage change from T1 to T2.

^b^ n = 48.

^c^ n = 29.

^d^ n = 7.

^e^ Average frequency of leaving home/day.

^f^ Average legs/day.

^g^ Average trips/day.

^h^ Average night trips/day.

##### Frequency of travel

At T2, 36 participants reported leaving their residence 589 times over 504 days, registering a total of 1209 trip legs. Increased travel frequency was observed at T2 post‐OTDA for all elements except night travel, which was observed to reduce by 13%.

##### Purpose of travel

A 13% increase in occupational participation occurred at T2. After applying OTPF 4th Ed classification of occupations (Boop et al., 2020) to the purpose of travel, instrumental activities of daily living (IADL) remained the most frequent motivation for travel (n = 200), although it reflected a 15% reduction in community‐access occasions from T1 to T2. Rates of participation increased for leisure (175%), work (23%), and social participation (21%) and reduced for engagement in health management occupations (50%).

##### Mode of travel

Except for the increased frequency of private car use, all other defined modes of transport recorded a reduction in use.

##### Travel companion

The presence of travel companions reduced across most groups except for the company of ‘friends’, which increased by 90% and dependent children (62% increase). The need for ‘driver assistance’ and ‘driver and other assistance’ reduced by 71% and 7%, respectively. Conversely, when one or more travel companion/s were present, this was primarily for social reasons (67%) and reflected 326% increase from T1 occasions.

##### Difficulty

Increased ease of community access performance was described at T2.

#### 3.1.4 Travel diary and driver status

On average, drivers participated in more occupations and travelled with greater frequency and less support than non‐drivers. Occasions of night travel increased at T2 for drivers (23%) and decreased for non‐drivers (−66.7%). IADL reflected a greater proportion of the reason for community access for non‐drivers at T2 (43%). An increased proportion of trips was associated with social participation (22%) and work (14%) for drivers, whereas non‐drivers reduced participation occasions by 50% and 78% at T2, respectively. Non‐drivers increased use of public transport by 48% and walking by 36% at T2 whereas drivers reduced use of all modes of transport with the exception of private vehicle use which increased (24%). When company was present and when dependent children were excluded, non‐drivers indicated a 57% increase in need for ‘driver and other assistance’ whereas drivers reported 597% increase in company only being present for social purposes.

### 3.2 Qualitative findings

Twelve participants (50% female), aged 42–64 years (mean = 54.6 years), with a diagnosis of stroke (n = 4), aneurysm (n = 4), TBI (n = 1), hypoxic brain injury (n = 1), and brain infection (n = 1), engaged in semi‐structured interviews at T2. Most participants resided in outer metropolitan (n = 7) and inner metropolitan (n = 4) areas, with only one participant living in a regional location according to remoteness classifications (Health & Welfare, 2004).

**FIGURE 5:**
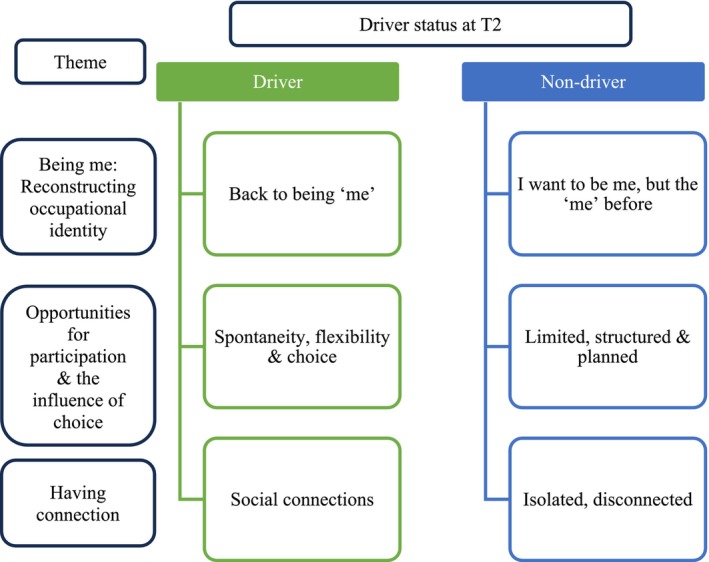
Qualitative themes.

Three overarching themes were inductively constructed to understand lifespace and occupational participation at T2 6 months post‐OTDA: (i) ‘Being me’—reconstructing occupational identity, (ii) opportunities for participation and the influence of choice, and (iii) ‘Having connection’ and impacts on wellbeing. Whereas themes illustrate common and meaningful patterns, the expression of these themes often contrasted according to driver status with drivers (n = 8) and non‐drivers (n = 4) experiencing different trajectories as illustrated in Figure 5. Pseudonyms have been used to preserve participant anonymity.

#### 3.2.1 Theme 1: ‘Being me’—reconstructing occupational identity

##### Drivers

Those who had returned to driving described the time following OTDA as a period of reclaiming their previously held occupational identity. A universal sense of ‘getting life back’ was described.

I'm enjoying my life a little bit right now, like I've got it back because it does take it away from you when you've got a brain injury. I didn't have the same house anymore, I didn't have a job anymore, I couldn't drive anymore. Everything in my life was turned upside down. But now I think, ‘I'll visit a friend’ … and I can go and do the groceries. Kelly (50 years, 23 months post aneurysm)

A return to driving enabled re‐engagement with previously valued occupations in a way that was not possible during driving disruption nor experienced by non‐drivers at T2. There was a shift in how drivers sensed themselves, transitioning away from burdening others with their dependency to being meaningful contributors in their own right. A rekindling of purpose was evident, positively influencing occupational identity.

I really got back to doing things like going to shopping, catching up with friends …doing all those parental tasks that were landing on the shoulders of my wife while I was in rehab. So taking kids to and from school, taking them to dancing lessons and music lessons and all that sort of stuff. But being able to contribute in a meaningful way… to the family. Joe (42 years, 13 months post hypoxic brain injury)

##### Non‐drivers

Whereas drivers associated a return to driving with a return to normalcy, non‐drivers continued to yearn for a return to their pre‐injury occupational self.

I want to be me, but me before … And the ‘me’ before was getting out and about, doing the volunteer work, talking … interacting with people. Madonna (62 years, 24 months post stroke)

The impact of occupational disruption persisted 6 months beyond OTDA. There was a level of resignation that current participation in community‐based occupations was unlikely to change and many non‐drivers were unable to imagine a future that included greater participation and engagement.

Oh well there's not much I can do about it is there? I can't drive a car, I can't do anything. If I [want to]go somewhere else, I can't go. If I [want to]do something, I can't go. I am stuck. Hai (59 years, 13 months post stroke)

Yet this was not unanimous among non‐drivers. For some, an acceptance of the impact of their ABI and their consequential inability to drive together with a strong desire to re‐engage in occupations motivated their adaptation.

I don't want people to look at me now and think, ‘This is who I am compared to that's who I was.’ I know that sounds silly … I'm not going to be [who I was], I know, but I'm trying to aim to get there, and get out and do things. Madonna (62 years, 24 months post stroke)

#### 3.2.2 Theme 2: Opportunities for participation and the influence of choice

##### Drivers

Spontaneity and flexibility characterised occupational participation opportunities for drivers. They reported engaging in a greater variety of community‐based endeavours with increased frequency. Drivers readily linked their capacity to drive with increased opportunities to participate in away‐from‐home occupations.

It's [return to driving] given my life back in many respects, actually. Or the opportunity to expand, to be able to go and do the things that I really like to do. Linda (62 years, 36 months post stroke)

Drivers perceived their post‐return to driving lifestyle as less structured and relished the flexibility and spontaneity which often underpinned their engagement in away‐from‐home occupations. Participants enjoyed being able to do things such as go ‘to the shops three times in one day’ *Linda (62 years, 36 months post stroke)*, change their mind in relation how they ordered their errands, or squeeze in a visit to a friend, reflecting an enhanced sense of control over what they elected to do with their time. A return to driving simultaneously offered expanded occupational choice possibilities while reducing the extensive pre‐planning required when dependent on others for community access.

Just to be able to literally go out my front door knowing that I can go out when I want to and where I want to is just fabulous. The freedom is just … there's no trapped feeling, there's no feeling like you've got to rely on other people; just that whole feeling of being a burden to people … I can drive myself to Pilates, I can drive myself to my friend's house, I can go out for coffee, I can do my own grocery shopping, I can go to doctor's appointments. It's huge. Sarah (46 years, 16 months post subarachnoid haemorrhage)

Finally, a change in how time was spent while in the community was identified. Drivers reflected on community access opportunities during driving disruption as being highly purposeful (e.g., attending an appointment or completing the shopping) with priority tasks being undertaken with efficiency in order to minimise the burden placed on those providing the transport and support. In contrast, a return to independent driving brought opportunity to linger in the experience, the opportunity to ‘just be’.

And then I just wander around all the shops… whereas before I probably would have just gone to the grocery shops and then straight home. Karen (52 years, 19 months post aneurysm)

##### Non‐drivers

The limited participation in away‐from‐home occupations reported by most non‐drivers denoted the structured and planned nature of participation. A perceived link between driving status and opportunities for engagement in community‐based occupations was identified.

It [not driving] just stops me getting a job… stops me doing a lot of things. I'm pretty much home 24/7. Michael (47 years, 20 months post TBI)

Non‐drivers acknowledged their constricted participation and attributed this to a range of factors. Difficulties identifying interests to pursue, a lack of alternative transport options, and financial constraints influenced capacity to engage in away‐from‐home occupations. Even when provisions had been made to accommodate non‐driver status, these were often insufficient to enable or maintain regular involvement in community‐based endeavours.

They [National Disability Insurance Scheme] give me a couple of bucks for Ubers. But you know that doesn't last long. Like I'm miles from anywhere so it costs a fortune to do any of that sort of stuff. So I just use the money to put extra fuel in the cars. An Uber down to the shops is $25. So it's either like go shopping once a week or put fuel in the car and somebody can go down and get it. Michael (47 years, 20 months post TBI)

When engagement in community‐based occupations did occur, it was primarily for IADL and health management, with limited instances of participation in work or social activities. Difficulties adjusting to the occupational challenges arising from ABI and/or not driving was often matched with low levels of motivation to change the status quo. Maladaptive strategies were employed and opportunities for participation were deflected.

I am most in the house … I send my carer to do it [shopping, taking daughter to school]. If I ask them to do things for me, they will do it. Sometimes we have a conversation and I will go with them. Kwame (64 years, 19 months post aneurysm)

Non‐drivers frequently described community‐access and participation opportunities as highly structured and often for the purpose of health management. Pre‐planning was required to organise formal support worker or family assistance to ensure attendance at appointments could occur. In recognition of the finite resources available to support engagement in away‐from‐home occupations, requests for assistance were rationed with leisure and social occupations less frequently considered.

I've got a driver who takes me to physio. I keep them just for appointments … I give them enough notice for them to do that. Kwame (64 years, 19 months post aneurysm)

#### 3.2.3 Theme 3: ‘Having connection’ and impacts on wellbeing

##### Drivers

Beyond general opportunities for participation, drivers acknowledged the amplified role social participation played in their reclaimed control over what they chose to do when leaving home. Being able to connect with friends was highly valued and positively impacted wellbeing.

I think I will go visit a friend, I couldn't do that before, I felt very isolated. You feel so alone. So driving is so important. Kelly (50 years, 23 months post aneurysm)

Being able to drive removed barriers to connecting with others in two ways. First, drivers were relieved of the burden of asking for and/or accepting the offers of support necessary to create social participation opportunities during driving disruption. Drivers no longer turned down invitations that had previously relied on others exceeding reasonable expectations to facilitate connection. Second, a return to driving shifted participants from being the recipient of support for transport and other needs to being the provider of support. Being able to give rather than receive support enhanced a sense of wellbeing for drivers.

She [Mother] had an injury, so I took her to her GP all the time and I could just do it easily, driving. I couldn't have done that before. And it was wonderful. It felt a lot better that I could actually care for someone else rather than being the one [cared for]. I was so grateful I could drive. Karen (52 years, 19 months post aneurysm)

##### Non‐drivers

Social and emotional impacts of occupational disruption persisted 6 months beyond OTDA. Participants reported limited social interaction and felt isolated and disconnected. They expressed turmoil, frustrated by their inability to drive, yet cognisant of potential consequences arising from driving when not ready to do so.

I don't want to talk anybody anymore … I can't handle [not driving] any more … I'd rather suffer myself [by not driving] than to kill someone for nothing. Hai (59 years, 13 months post stroke)

Often the strategies initiated by health and support services to compensate for a participants' non‐driving status accentuated social isolation and further limited opportunities for social interactions. For instance, tele‐health replaced face to face appointments. Whereas this strategy enabled important health consultations to occur and alleviated demand for transport to be arranged, it reduced the opportunity to build more meaningful connections, potentially contributing to a sense of isolation. Likewise, the benefits of formal support services being delivered in‐home limited social connection to formal networks and diminished opportunities for casual interaction.

I get people to come here now that I'm on the NDIS … I've got no friends. And me [sic] family's too far away. Michael (47 years, 20 months post TBI)

Some non‐drivers recognised the importance of social connection to their wellbeing. They valued participating in the social networking opportunities that comprised their transitional rehabilitation programme and were disappointed not to be able to continue engagement in such time‐limited programmes. However, some participants created opportunities for casual interactions and more meaningful connections.

I've started interacting with the community more. I've started to volunteering[sic]. Getting out and about, doing the volunteer work, talking, chatting, laughing just interacting with people… Having an interesting conversation that was more than ‘Good morning, how are you?’ Madonna (62 years, 24 months post stroke)

#### 3.2.4 Integrated findings

The integrated quantitative and qualitative data set results concerning the trajectory of lifespace and occupational participation following ABI from pre‐OTDA (T1) to 6 months post‐OTDA are presented in Table 4. Triangulation of the data sources and timepoint comparisons confirmed lifespace and occupational participation following OTDA were influenced by the outcome of the OTDA process.

**TABLE 4:** Integrated analysis.

Theme	LSMA‐C		Travel diary		Interview	
	Driver	Non‐driver	Driver	Non‐driver	Driver	Non‐driver
**Where: ‘Opportunities for participation’** **Access to community** ≥ 4 times/week: % (trajectory)	**Average total LSMA‐C score** (trajectory)		**Average number of times participant left home/day** (trajectory)		‘From a driving perspective, I'm pretty much, I hate to use that word but … but back to normal. We both work, so we drive autonomously through the week and then on the weekends I'll jump in the car… We're about to head down the coast on holidays, so it's an hour's drive. It's just pretty much the same as we've always done.’ *Andrew*	‘I am most in the house and sometimes I go out with my [support] worker, just do some exercise. When the weather is good but if the weather is no good I don't do it.’ *Kwame*
	79.4 (↑ 35%)	46.1 (↓ 20%)	1.2 (↑ 22%)	1.05 (↑ 27%)		
i Local neighbourhood (zone 3)	90.3 (↑ 8%)	57.1 (↓ 43%)				
ii Within city (zone 4)	77.4 (↑ 71%)	28.6 (↓ 71%)				
iii Beyond city (zone 5)	19.4 (↑ 198%)	0 (no change)				
**Why** **‘Being me’** Most frequent **reasons for leaving home,** % (trajectory)			IADL 31.3% (↓ 22%)	i IADL 43.2% (↑ 6%)	‘[Return to driving] has given my life back… to be able to go and do the things that I really like to do.’ *Linda*	‘If I can't drive a car, I can't do anything.’ *Kwame*
			ii Social participation 22.0% (↑ 16%)	ii Leisure 23.5% (↑ 19%)		
			iii Leisure 20.9% (↑ 273%)	iii Health management 22.2% (↓ 17%)		
			iv Work 13.8% (↑ 82%)	iv Social participation 9.9% (↓ 50%)		
			v Health management 11.8% (↓ 57%)	v Work 1.2% (↓ 78%)		
**With whom** **‘Having connection’**	**Support to access community** n (%)		**Level of reliance**: At least one companion on one or more trip legs/day n (%, trajectory)		‘I have visited friends' places more since I can drive, because before I would feel bad that they'd say, “I'll come and pick you up,” but I'd feel like, no… I think I get more invitations, actually. Maybe, because it's just like, you'll be right to get here, yep. I get more opportunities because it's just more normal or something. I can just fit in with everybody.’ *Karen*	‘And that's what I've missed most of all; just connecting with people. Especially the initial stages, people ring to talk to you and what not, but as time goes on, and life takes over, of course that drops off.’ *Donna*
i Local neighbourhood (zone 3)	2 (7%)	6 (86%)	150 (37, ↓ 46%)	37 (36, ↓ 22%)		
ii Within city (zone 4)	4 (13%)	6 (86%)	**Purpose of companion/s:** Driver only			
iii Beyond city (zone 5)	7 (23%)	7(100%)	10 (10, ↓ 87%)	18 (51, ↓ 22%)		
			Driver and other support			
			6 (8, ↓ 39%)	9 (26, ↑ 57%)		
			Social only			
			83 (82, ↑ 597%)	8 (23, ↑ 30%)		
**How** **Mode of transport** n (%):			Private car		‘I'm free to travel wherever I like now, whereas before, if I wanted to go anywhere that wasn't on public transport, I had to use my spouse. Because now I'm driving I don't have to tell him when I'm going and give him 6 months notice.’ *Susan*	‘Most of them [health appointments] are telehealth or I get people to come here now that I'm on the NDIS.’ *Michael*
			274 (72, ↑ 24%)	40 (62, ↑ 1%)		
			Walking			
			71(18.6, ↓ 33%)	16 (24.6, ↑ 36%)		
			Public transport			
			18 (4.7, ↓ 16%)	8 (12.3, ↑ 48%)		

For those who had returned to driving post‐OTDA, the achieved increase in lifespace and occupational participation was corroborated by all three data elements. The average LSMA‐C score for drivers (79.4) indicates an extended lifespace, supported by increased access to all zones beyond the place of residence. The positive influence of independent driving on participation, described during in‐depth interviews, complemented travel diary findings illustrating increased frequency of leaving home, greater engagement in a wider range of community‐based occupations, reduced reliance on support, and increased social participation.

A contracted lifespace with reduced levels of occupational participation was observed across non‐driver LSMA‐C scores, travel diary outcomes, and interview findings. Results indicate occasions of community access occurred less frequently, within a more limited geographical distance from home, and with greater dependency on others than drivers. In addition to being below the score indicative of restricted lifespace (≤60), LSMA‐C total score at T2 (mean = 46.1) was lower than scores achieved by this group at T1 (mean = 57.3) indicating a deteriorating lifespace. These results align with interview findings reporting reduced opportunities to participate in away‐from‐home occupations (e.g., having others undertake community‐based occupations such as shopping), increased delivery of services within home (e.g., telehealth), and travel diary learnings, which reflect a greater reliance on support for transport and other needs by non‐drivers.

## 4 DISCUSSION

This is the first study to investigate the trajectory of lifespace and occupational participation following an OTDA. Findings indicate the outcome of OTDA, that is, whether participants do or do not return to independent driving, influences the extent to which participants with an ABI access and participate in community‐based occupations. Whereas drivers experienced an increase in flexible and spontaneous engagement in away‐from‐home occupations, non‐drivers reported a restricted lifespace, marked by structured and less frequent opportunities for participation that were largely purpose‐driven and reliant on others to facilitate.

Contrary to evidence supporting a persistent and progressively deteriorating lifespace over the 24 months following stroke for older adults (Tsunoda et al., 2022), improved lifespace was achieved in this study by those who had returned to driving. Drivers accessed places beyond their residence to a greater extent than non‐drivers, simultaneously reducing reliance on others while extending their community access. The restricted lifespace realised by non‐drivers in this study was more limited than the lifespace of older non‐drivers (Hashimoto, 2021) yet conformed to the diminishing lifespace trajectory observed for older persons who experienced stroke (Tsunoda et al., 2022). It is unclear what will transpire for non‐drivers following the abrupt decline in lifespace observed 6 months post‐OTDA. A progressive gradual decline in lifespace may eventuate in the manner experienced by older adults following driving cessation (Huisingh et al., 2017). Alternatively, the desire to engage and participate in community‐based occupations post‐ABI may enhance occupational adaptation processes (Klinger, 2005) resulting in a steadying or improved lifespace. Further exploration is required.

Previous identification of driving status as a significant predictor of participation following TBI (Erler et al., 2018) and stroke (White et al., 2012) was reinforced in this broader ABI study exploring occupational participation. As evident in travel diary findings, participants who had returned to driving at T2 reported an 18% increase in occupational participation demonstrating an expansion in their capacity to initiate, access, and sustain engagement in community‐based occupations (Egan & Restall, 2022). A return to driving removed an environmental barrier to engagement experienced following ABI during driving disruption (Gingrich et al., 2023). Conversely, a deteriorating pre‐ to post‐OTDA occupational participation trajectory (−9%) was experienced by non‐drivers who often reported being ‘stuck at home’ and unable to imagine future capacity for engagement in occupations away‐from‐home. Findings reinforce the notion that occupational participation and engagement facilitate the occupational adaptation process necessary to maximise community reintegration and wellbeing following TBI (Klepo et al., 2022; Klinger, 2005) and stroke (Wassenius et al., 2022). Interestingly, health management was the only domain where reduced participation was evident for both drivers and non‐drivers at T2. This may indicate the exploration of capacity to return to driving as one of the final steps in rehabilitation programmes. It may reflect a time when the referring doctor has deemed sufficient recovery to have occurred not only to open the possibility of return to driving but potentially to indicate reduced need for ongoing rehabilitation services. Yet findings indicate the period following OTDA, particularly for those unsuccessful with their return to driving goal, may be a time to focus on interventions that foster community access skills, habits, and routines that build the competency, confidence, acceptance of new self, and consequent occupational adaptation necessary for participation in community‐based occupations following ABI (Parsons & Stanley, 2008).

The restoration of choice and control in decision making has been described as a dynamic although potentially challenging process following ABI (Murray et al., 2022; Ownsworth et al., 2024). A return to driving goal and participation in OTDA signals a desire to re‐engage in this occupation and to extend transport choices for community access. For those who had resumed driving, a reclaiming of agency was observed, with those demonstrating driving competency empowered to engage in more varied away‐from‐home pursuits with greater spontaneity and flexibility in decision making. In contrast, non‐drivers often struggled with the discrepancy between their desire to take control with occupations linked with driving and their assessed inability to do so. Two features identified by Walder and Molineux (2017) as detrimental to the reconstruction of occupational identity post‐chronic illness or injury were noted for many non‐drivers. First, a lack of motivation to initiate or engage in community‐based occupations was identified. Second, insufficient competency or confidence accessing their community was reported. Clinicians should seek to understand reasons for non‐participation in community‐based occupations in order to extend choice and control opportunities and to address changes to occupational identity post‐ABI for non‐drivers.

The ABI sequelae preventing a return to driving may also compromise skills and capacity to regain agency and exert choice, compounding difficulties. On the other hand, it may be argued that non‐drivers in this study were able to execute their choice in preferencing support workers to complete IADL and other community‐based occupations on their behalf rather than pursuing avenues to enable their participation. Further understanding of the reasons underpinning this decision making is required to better comprehend findings. Whereas some participants may have little desire to participate in a range of community‐based occupations (e.g., they may preference home‐based occupations such as crafting, online gaming etc), it is more likely that multiple factors are at play. In addition to ABI‐related changes (Kersey et al., 2020), changes to occupational participation (Olofsson et al., 2017) and occupational identity (Bassingthwaighte et al., 2025) may impact decision making with maladaptive adjustment to occupational challenges occurring (de Graaf et al., 2022; Klepo et al., 2022), compounding a loss of agency. For those unsuccessful in returning to driving following OTDA, goal setting and therapy aiming to create, enhance, and support occupational choice and control may facilitate a graduated return to agency (Ownsworth et al., 2024), enhancing occupational participation and occupational identity and providing an alternate mechanism to consider the ‘wicked’ problem of driving disruption (Varpio et al., 2017).

This study reaffirms recent findings that reduced social participation and constricted opportunities for connection are experienced following TBI (Leeson et al., 2024) and stroke (Ianni et al., 2023) and larger lifespace is associated with greater levels of overall social and community engagement for older adults (Mauldin, 2024). This study, where driver status influenced opportunity for social participation and connection, broadens the association to include adults following ABI. When considering the ‘why’ of community access, those returning to driving elected to increase their participation in social occupations at T2. They actively sought and relished greater opportunities for social connection, confirming the positive association between social connection and wellbeing following TBI (Leeson et al., 2024). The occupational participation profile of non‐drivers captured in travel diary findings at T2 indicated an increased predominance of IADL occupations and fading social participation with a concurrent reduction in the presence of company when accessing the community. This may indicate some non‐drivers had developed competence in accessing their community independently (e.g., public transport, walking), yet the focus on completing ‘essential’ IADL and the time and effort required to do so may have diminished capacity and time availability to engage in social occupations. Furthermore, the reduced level of support (primarily provided by family members pre‐OTDA) to complete away‐from‐home occupations also resulted in reduced opportunity for social connection. Given social participation in neighbourhood community has been found to prevent onset of disability for older people with limited lifespace (Fujii, 2024), exploration of opportunities to increase participation in social occupations following ABI may be indicated.

This study has identified several concepts for future exploration. First, it is unclear why the lifespace and frequency of occupational participation deteriorated over the 6 months post‐OTDA for participants who had not returned to driving at T2. Further understanding of the availability of, engagement with, and nature of rehabilitation and support services post‐OTDA may shape the delivery of services to better meet client needs. Second, defining and investigating the relationship between lifespace and participation in community‐based occupations following ABI more broadly may enhance occupational participation outcomes. Finally, exploration of interventions to facilitate increased access to and sustained participation in community‐based occupations following ABI is indicated, particularly for those unsuccessful with their return to driving goal post‐OTDA.

There are several notable limitations of this study to be considered. First, with the exception of COVID‐related reasons, this study did not explore why participants did not leave home. Understanding this context would clarify the extent choice played in stay‐at‐home occasions to better understand barriers and enablers to community‐based participation. Second, information regarding participant living situation (e.g., living alone, with family, with friends) was not collected. Being able to appreciate the presence or otherwise of household occupants and their relationship with participants may identify factors that influence participation in away‐from‐home occupations. Finally, the cohort for this study was individuals who had been referred for OTDA following ABI at one Australian metropolitan hospital. Findings may not generalise to non‐drivers post‐ABI who have not been determined as ready to participate in an OTDA, nor to those living in more regional or remote locations. Given the high reliance on driving as a means to access community in Australia, findings may not translate to other contexts where dependence on driving for transport is not as dominant.

## 5 CONCLUSION

This study found that after ABI, the trajectory of lifespace, and participation in community‐based occupations pre‐ to post‐OTDA was influenced by driver status. Drivers experienced increased lifespace with greater opportunities to control participation in community‐based occupations with flexibility and spontaneity, whereas non‐drivers left home less frequently primarily to undertake structured IADL occupations. These findings suggest that the conduct of OTDA, particularly for those unable to return to driving, marks a time when additional services and supports are required to enhance engagement in valued away‐from‐home occupations to promote occupational adaptation, facilitate the graduated return of agency, and enhance community reintegration outcomes.

## AUTHOR CONTRIBUTIONS

All authors made substantial contributions to the concept (L. B., L. G., and M. M.), design (L. B., L. G., and M. M.), acquisition (L. B.), analysis (L. B., L. G., and M. M.), or interpretation (L. B., L. G., and M. M.) of data for the work; drafted (L. B.) or critically reviewed (L. G. and M. M.) intellectual content; approved the submitted version (L. B., L. G., and M. M.); and agreed to be accountable for all aspects of the work (L. B., L. G., and M. M.).

## CONFLICT OF INTEREST STATEMENT

One of the co‐authors, Louise Gustafsson, is the current Editor‐in‐Chief of *Australian Occupational Therapy Journal*. Louise Gustafsson was excluded from the peer review process, management of the peer review process and all decision‐making for this article as undertaken independently by an associate editor. The remaining authors have no other conflicts of interest to note.

## Supporting information


**Appendix S1.** Travel diary.

**Appendix S2** Point‐biserial correlation.

## Data Availability

The data that support the findings of this study are available on request from the corresponding author. The data are not publicly available due to privacy or ethical restrictions.
