# Supplementary material for: Changes in lifespace and participation in community‐based occupations of people with acquired brain injury: A mixed methods exploration 6 months following occupational therapy driving assessment
Source: Aust Occup Ther J. 2025 Apr 13;72(2):e70017. doi: 10.1111/1440-1630.70017 (PMC11994898; doi:10.1111/1440-1630.70017)
Supplement: Supplementary file 1 — Appendix S1. Travel diary. [file AOT-72-0-s001.docx]

**Travel Diary: Instructions**

Thank you for participating in this research. We are looking to understand how people get about their communities and engage in activities that are important to them after brain injury.

- We ask you to complete the following travel diary for each day over the next 14 days.
- You can complete the travel diary by yourself or with the assistance of a carer or family member.
- You may wish to write down each trip as you go or you may wish to sit down at the end of each day to record your activities. It is important to try to complete the travel diary at least on a daily basis to make sure all trips are recalled.
- There is a separate sheet for each day. If you travel to more destinations or have more information, please write the date and add details on the spare sheets at the back.
- A member of the research team will collect the documents at the conclusion of the 2 week period.
- If you have any questions about the travel diary, please call Louise Bassingthwaighte on 3176 5008.

**TRAVEL DIARY** Week One: Day of Week:_______________ Date:___________

| **Time of Travel** | **Where?** | **Why?** | **How?** | **With Whom?** | **How easy was the trip?** | **Comments** |
| --- | --- | --- | --- | --- | --- | --- |
| What time did you start and end your trip? E.g. 10am – noon. | Where did you go?  E.g. From home to Chermside Shopping Centre  E.g. From mates home in West End to SouthBank | What was the purpose of the trip? Eg. social event, work, shopping, errands, appointments, volunteering etc | How did you get to your destination?  E.g. Walking, bus, train, lift with friend, lift with family etc. You may have used more than 1 way to get somewhere. | Did you travel alone or did you have someone with you?  E.g. Family member  If you travelled with someone, did they assist you? What did they do? e.g. driver, help to get on the bus | Rate your trip:   - Very Easy - Easy - Not easy or hard - Difficult - Very Difficult |  |
|  |  |  |  |  |  |  |
|  |  |  |  |  |  |  |
|  |  |  |  |  |  |  |
|  |  |  |  |  |  |  |

**TRAVEL DIARY Week One: Day of Week:_______________ Date:___________**

| **Time of Travel** | **Where?** | **Why?** | **How?** | **With Whom?** | **How easy was the trip?** | **Comments** |
| --- | --- | --- | --- | --- | --- | --- |
| What time did you start and end your trip? E.g. 10am – noon. | Where did you go?  E.g. From home to Chermside Shopping Centre  E.g. From mates home in West End to SouthBank | What was the purpose of the trip? Eg. social event, work, shopping, errands, appointments, volunteering etc | How did you get to your destination?  E.g. Walking, bus, train, lift with friend, lift with family etc. You may have used more than 1 way to get somewhere. | Did you travel alone or did you have someone with you?  E.g. Family member  If you travelled with someone, did they assist you? What did they do? e.g. driver, help to get on the bus | Rate your trip:   - Very Easy - Easy - Not easy or hard - Difficult - Very Difficult | Anything to note? |
|  |  |  |  |  |  |  |
|  |  |  |  |  |  |  |
|  |  |  |  |  |  |  |
|  |  |  |  |  |  |  |

**TRAVEL DIARY Week One: Day of Week:_______________ Date:___________**

| **Time of Travel** | **Where?** | **Why?** | **How?** | **With Whom?** | **How easy was the trip?** | **Comments** |
| --- | --- | --- | --- | --- | --- | --- |
| What time did you start and end your trip? E.g. 10am – noon. | Where did you go?  E.g. From home to Chermside Shopping Centre  E.g. From mates home in West End to SouthBank | What was the purpose of the trip? Eg. social event, work, shopping, errands, appointments, volunteering etc | How did you get to your destination?  E.g. Walking, bus, train, lift with friend, lift with family etc. You may have used more than 1 way to get somewhere. | Did you travel alone or did you have someone with you?  E.g. Family member  If you travelled with someone, did they assist you? What did they do? e.g. driver, help to get on the bus | Rate your trip:   - Very Easy - Easy - Not easy or hard - Difficult - Very Difficult | Anything to note? |
|  |  |  |  |  |  |  |
|  |  |  |  |  |  |  |
|  |  |  |  |  |  |  |
|  |  |  |  |  |  |  |

**TRAVEL DIARY Week One: Day of Week:_______________ Date:___________**

| **Time of Travel** | **Where?** | **Why?** | **How?** | **With Whom?** | **How easy was the trip?** | **Comments** |
| --- | --- | --- | --- | --- | --- | --- |
| What time did you start and end your trip? E.g. 10am – noon. | Where did you go?  E.g. From home to Chermside Shopping Centre  E.g. From mates home in West End to SouthBank | What was the purpose of the trip? Eg. social event, work, shopping, errands, appointments, volunteering etc | How did you get to your destination?  E.g. Walking, bus, train, lift with friend, lift with family etc. You may have used more than 1 way to get somewhere. | Did you travel alone or did you have someone with you?  E.g. Family member  If you travelled with someone, did they assist you? What did they do? e.g. driver, help to get on the bus | Rate your trip:   - Very Easy - Easy - Not easy or hard - Difficult - Very Difficult | Anything to note? |
|  |  |  |  |  |  |  |
|  |  |  |  |  |  |  |
|  |  |  |  |  |  |  |
|  |  |  |  |  |  |  |

**TRAVEL DIARY Week One: Day of Week:_______________ Date:___________**

| **Time of Travel** | **Where?** | **Why?** | **How?** | **With Whom?** | **How easy was the trip?** | **Comments** |
| --- | --- | --- | --- | --- | --- | --- |
| What time did you start and end your trip? E.g. 10am – noon. | Where did you go?  E.g. From home to Chermside Shopping Centre  E.g. From mates home in West End to SouthBank | What was the purpose of the trip? Eg. social event, work, shopping, errands, appointments, volunteering etc | How did you get to your destination?  E.g. Walking, bus, train, lift with friend, lift with family etc. You may have used more than 1 way to get somewhere. | Did you travel alone or did you have someone with you?  E.g. Family member  If you travelled with someone, did they assist you? What did they do? e.g. driver, help to get on the bus | Rate your trip:   - Very Easy - Easy - Not easy or hard - Difficult - Very Difficult | Anything to note? |
|  |  |  |  |  |  |  |
|  |  |  |  |  |  |  |
|  |  |  |  |  |  |  |
|  |  |  |  |  |  |  |

**TRAVEL DIARY Week One: Day of Week:_______________ Date:___________**

| **Time of Travel** | **Where?** | **Why?** | **How?** | **With Whom?** | **How easy was the trip?** | **Comments** |
| --- | --- | --- | --- | --- | --- | --- |
| What time did you start and end your trip? E.g. 10am – noon. | Where did you go?  E.g. From home to Chermside Shopping Centre  E.g. From mates home in West End to SouthBank | What was the purpose of the trip? Eg. social event, work, shopping, errands, appointments, volunteering etc | How did you get to your destination?  E.g. Walking, bus, train, lift with friend, lift with family etc. You may have used more than 1 way to get somewhere. | Did you travel alone or did you have someone with you?  E.g. Family member  If you travelled with someone, did they assist you? What did they do? e.g. driver, help to get on the bus | Rate your trip:   - Very Easy - Easy - Not easy or hard - Difficult - Very Difficult | Anything to note? |
|  |  |  |  |  |  |  |
|  |  |  |  |  |  |  |
|  |  |  |  |  |  |  |
|  |  |  |  |  |  |  |

**TRAVEL DIARY Week One:** Day of Week:_______________ Date:___________

| **Time of Travel** | **Where?** | **Why?** | **How?** | **With Whom?** | **How easy was the trip?** | **Comments** |
| --- | --- | --- | --- | --- | --- | --- |
| What time did you start and end your trip? Eg. 10am-noon | Where did you go?  Eg. From home to Chermside Shopping Centre  Eg. From mates home in West End to SouthBank | What was the purpose of the trip? Eg. social event, work, shopping, errands, appointments, volunteering etc | How did you get to your destination?  Eg. Walking, bus, train, lift with friend, lift with family etc. You may have used more than 1 way to get somewhere. | Did you travel alone or did you have someone with you?  Eg. Family member  If you travelled with someone, did they have a job eg driver, help to get on the bus | Rate your trip:   - Very Easy - Easy - Not easy or hard - Difficult - Very Difficult | Anything to note? |
|  |  |  |  |  |  |  |
|  |  |  |  |  |  |  |
|  |  |  |  |  |  |  |
|  |  |  |  |  |  |  |

**TRAVEL DIARY Week Two:** Day of Week:_______________ Date:___________

| **Time of Travel** | **Where?** | **Why?** | **How?** | **With Whom?** | **How easy was the trip?** | **Comments** |
| --- | --- | --- | --- | --- | --- | --- |
| What time did you start and end your trip? Eg. 10am-noon | Where did you go?  Eg. From home to Chermside Shopping Centre  Eg. From mates home in West End to SouthBank | What was the purpose of the trip? Eg. social event, work, shopping, errands, appointments, volunteering etc | How did you get to your destination?  Eg. Walking, bus, train, lift with friend, lift with family etc. You may have used more than 1 way to get somewhere. | Did you travel alone or did you have someone with you?  Eg. Family member  If you travelled with someone, did they have a job eg driver, help to get on the bus | Rate your trip:   - Very Easy - Easy - Not easy or hard - Difficult - Very Difficult | Anything to note? |
|  |  |  |  |  |  |  |
|  |  |  |  |  |  |  |
|  |  |  |  |  |  |  |
|  |  |  |  |  |  |  |

**TRAVEL DIARY Week Two:** Day of Week:_______________ Date:___________

| **Time of Travel** | **Where?** | **Why?** | **How?** | **With Whom?** | **How easy was the trip?** | **Comments** |
| --- | --- | --- | --- | --- | --- | --- |
| What time did you start and end your trip? Eg. 10am-noon | Where did you go?  Eg. From home to Chermside Shopping Centre  Eg. From mates home in West End to SouthBank | What was the purpose of the trip? Eg. social event, work, shopping, errands, appointments, volunteering etc | How did you get to your destination?  Eg. Walking, bus, train, lift with friend, lift with family etc. You may have used more than 1 way to get somewhere. | Did you travel alone or did you have someone with you?  Eg. Family member  If you travelled with someone, did they have a job eg driver, help to get on the bus | Rate your trip:   - Very Easy - Easy - Not easy or hard - Difficult - Very Difficult | Anything to note? |
|  |  |  |  |  |  |  |
|  |  |  |  |  |  |  |
|  |  |  |  |  |  |  |
|  |  |  |  |  |  |  |

**TRAVEL DIARY Week Two:** Day of Week:_______________ Date:___________

| **Time of Travel** | **Where?** | **Why?** | **How?** | **With Whom?** | **How easy was the trip?** | **Comments** |
| --- | --- | --- | --- | --- | --- | --- |
| What time did you start and end your trip? Eg. 10am-noon | Where did you go?  Eg. From home to Chermside Shopping Centre  Eg. From mates home in West End to SouthBank | What was the purpose of the trip? Eg. social event, work, shopping, errands, appointments, volunteering etc | How did you get to your destination?  Eg. Walking, bus, train, lift with friend, lift with family etc. You may have used more than 1 way to get somewhere. | Did you travel alone or did you have someone with you?  Eg. Family member  If you travelled with someone, did they have a job eg driver, help to get on the bus | Rate your trip:   - Very Easy - Easy - Not easy or hard - Difficult - Very Difficult | Anything to note? |
|  |  |  |  |  |  |  |
|  |  |  |  |  |  |  |
|  |  |  |  |  |  |  |
|  |  |  |  |  |  |  |

**TRAVEL DIARY Week Two:** Day of Week:_______________ Date:___________

| **Time of Travel** | **Where?** | **Why?** | **How?** | **With Whom?** | **How easy was the trip?** | **Comments** |
| --- | --- | --- | --- | --- | --- | --- |
| What time did you start and end your trip? Eg. 10am-noon | Where did you go?  Eg. From home to Chermside Shopping Centre  Eg. From mates home in West End to SouthBank | What was the purpose of the trip? Eg. social event, work, shopping, errands, appointments, volunteering etc | How did you get to your destination?  Eg. Walking, bus, train, lift with friend, lift with family etc. You may have used more than 1 way to get somewhere. | Did you travel alone or did you have someone with you?  Eg. Family member  If you travelled with someone, did they have a job eg driver, help to get on the bus | Rate your trip:   - Very Easy - Easy - Not easy or hard - Difficult - Very Difficult | Anything to note? |
|  |  |  |  |  |  |  |
|  |  |  |  |  |  |  |
|  |  |  |  |  |  |  |
|  |  |  |  |  |  |  |

**TRAVEL DIARY Week Two:** Day of Week:_______________ Date:___________

| **Time of Travel** | **Where?** | **Why?** | **How?** | **With Whom?** | **How easy was the trip?** | **Comments** |
| --- | --- | --- | --- | --- | --- | --- |
| What time did you start and end your trip? Eg. 10am-noon | Where did you go?  Eg. From home to Chermside Shopping Centre  Eg. From mates home in West End to SouthBank | What was the purpose of the trip? Eg. social event, work, shopping, errands, appointments, volunteering etc | How did you get to your destination?  Eg. Walking, bus, train, lift with friend, lift with family etc. You may have used more than 1 way to get somewhere. | Did you travel alone or did you have someone with you?  Eg. Family member  If you travelled with someone, did they have a job eg driver, help to get on the bus | Rate your trip:   - Very Easy - Easy - Not easy or hard - Difficult - Very Difficult | Anything to note? |
|  |  |  |  |  |  |  |
|  |  |  |  |  |  |  |
|  |  |  |  |  |  |  |
|  |  |  |  |  |  |  |

**TRAVEL DIARY Week Two:** Day of Week:_______________ Date:___________

| **Time of Travel** | **Where?** | **Why?** | **How?** | **With Whom?** | **How easy was the trip?** | **Comments** |
| --- | --- | --- | --- | --- | --- | --- |
| What time did you start and end your trip? Eg. 10am-noon | Where did you go?  Eg. From home to Chermside Shopping Centre  Eg. From mates home in West End to SouthBank | What was the purpose of the trip? Eg. social event, work, shopping, errands, appointments, volunteering etc | How did you get to your destination?  Eg. Walking, bus, train, lift with friend, lift with family etc. You may have used more than 1 way to get somewhere. | Did you travel alone or did you have someone with you?  Eg. Family member  If you travelled with someone, did they have a job eg driver, help to get on the bus | Rate your trip:   - Very Easy - Easy - Not easy or hard - Difficult - Very Difficult | Anything to note? |
|  |  |  |  |  |  |  |
|  |  |  |  |  |  |  |
|  |  |  |  |  |  |  |
|  |  |  |  |  |  |  |

**TRAVEL DIARY Week Two:** Day of Week:_______________ Date:___________

| **Time of Travel** | **Where?** | **Why?** | **How?** | **With Whom?** | **How easy was the trip?** | **Comments** |
| --- | --- | --- | --- | --- | --- | --- |
| What time did you start and end your trip? Eg. 10am-noon | Where did you go?  Eg. From home to Chermside Shopping Centre  Eg. From mates home in West End to SouthBank | What was the purpose of the trip? Eg. social event, work, shopping, errands, appointments, volunteering etc | How did you get to your destination?  Eg. Walking, bus, train, lift with friend, lift with family etc. You may have used more than 1 way to get somewhere. | Did you travel alone or did you have someone with you?  Eg. Family member  If you travelled with someone, did they have a job eg driver, help to get on the bus | Rate your trip:   - Very Easy - Easy - Not easy or hard - Difficult - Very Difficult | Anything to note? |
|  |  |  |  |  |  |  |
|  |  |  |  |  |  |  |
|  |  |  |  |  |  |  |
|  |  |  |  |  |  |  |
