# Supplementary material for: Changes in lifespace and participation in community‐based occupations of people with acquired brain injury: A mixed methods exploration 6 months following occupational therapy driving assessment
Source: Aust Occup Ther J. 2025 Apr 13;72(2):e70017. doi: 10.1111/1440-1630.70017 (PMC11994898; doi:10.1111/1440-1630.70017)
Supplement: Supplementary file 2 — Appendix S2 Point‐biserial correlation. [file AOT-72-0-s002.docx]

Supplementary Appendix B: Point-biserial correlation

|  | Outlier included | Outlier excluded |
| --- | --- | --- |
| Driving Status, n (%)  Driving  Not driving | 31(81.6)  7 (18.4) | 30 (81.1)  7 (18.9) |
| Point-biserial correlation | r_pb_(36)= -0.620, p<0.001 | r_pb_(35)= -0.664, p<0.001 |
| Lifespace, m (SD)  Driving  Not driving | 79.39 (17.83)  46.14 (9.75) | 80.67 (16.62)  46.14 (9.75) |
| Tests of Normality – Shapiro-Wilk Test:  Driving  Not driving | p = 0.185  p = 0.956 | p = 0.167  p = 0.956 |
| Test of Homogeneity of Variance – Levene Statistic | p = 0.823 | p = 0.099 |
| Magnitude of relationship (Cohen, 1988) | Strong (ie r>0.5) | Strong (ie r>0.5) |
| Coefficient of determination (Sheskin, 2011) | r_pb_^2^(36) = 38.44% | r_pb_^2^(36) = 44.08% |
